# Inhibition of neutrophil extracellular trap formation attenuates NLRP1-dependent neuronal pyroptosis *via* STING/IRE1α pathway after traumatic brain injury in mice

**DOI:** 10.3389/fimmu.2023.1125759

**Published:** 2023-04-14

**Authors:** Yiyao Cao, Mingming Shi, Liang Liu, Yan Zuo, Haoran Jia, Xiaobin Min, Xilei Liu, Zhijuan Chen, Yuan Zhou, Shenghui Li, Guili Yang, Xiao Liu, Quanjun Deng, Fanglian Chen, Xin Chen, Shu Zhang, Jianning Zhang

**Affiliations:** ^1^ Department of Neurosurgery, Tianjin Medical University General Hospital, Tianjin, China; ^2^ Key Laboratory of Post-trauma Neuro-repair and Regeneration in Central Nervous System, Tianjin Neurological Institute, Ministry of Education, Tianjin, China; ^3^ Tianjin Key Laboratory of Injuries, Variations and Regeneration of Nervous System, Tianjin Neurological Institute, Tianjin, China; ^4^ Department of Geriatrics, Tianjin Medical University General Hospital, Tianjin, China; ^5^ Baodi Clinical College, Tianjin Medical University, Tianjin, China

**Keywords:** neutrophil extracellular trap, traumatic brain injury, pyroptosis, NLRP1, STING, IRE1α

## Abstract

**Introduction:**

Increased neutrophil extracellular trap (NET) formation has been reported to be associated with cerebrovascular dysfunction and neurological deficits in traumatic brain injury (TBI). However, the biological function and underlying mechanisms of NETs in TBI-induced neuronal cell death are not yet fully understood.

**Methods:**

First, brain tissue and peripheral blood samples of TBI patients were collected, and NETs infiltration in TBI patients was detected by immunofluorescence staining and Western blot. Then, a controlled cortical impact device was used to model brain trauma in mice, and Anti-Ly6G, DNase, and CL-amidine were given to reduce the formation of neutrophilic or NETs in TBI mice to evaluate neuronal death and neurological function. Finally, the pathway changes of neuronal pyroptosis induced by NETs after TBI were investigated by administration of peptidylarginine deiminase 4 (a key enzyme of NET formation) adenovirus and inositol-requiring enzyme-1 alpha (IRE1α) inhibitors in TBI mice.

**Results:**

We detected that both peripheral circulating biomarkers of NETs and local NETs infiltration in the brain tissue were significantly increased and had positive correlations with worse intracranial pressure (ICP) and neurological dysfunction in TBI patients. Furthermore, the depletion of neutrophils effectively reduced the formation of NET in mice subjected to TBI. we found that degradation of NETs or inhibition of NET formation significantly inhibited nucleotide-binding oligomerization domain (NOD)-like receptor pyrin domain containing 1 (NLRP1) inflammasome-mediated neuronal pyroptosis after TBI, whereas these inhibitory effects were abolished by cyclic GMP-AMP (cGAMP), an activator of stimulating Interferon genes (STING). Moreover, overexpression of PAD4 in the cortex by adenoviruses could aggravate NLRP1-mediated neuronal pyroptosis and neurological deficits after TBI, whereas these pro-pyroptotic effects were rescued in mice also receiving STING antagonists. Finally, IRE1α activation was significantly upregulated after TBI, and NET formation or STING activation was found to promote this process. Notably, IRE1α inhibitor administration significantly abrogated NETs-induced NLRP1 inflammasome-mediated neuronal pyroptosis in TBI mice.

**Discussion:**

Our findings indicated that NETs could contribute to TBI-induced neurological deficits and neuronal death by promoting NLRP1-mediated neuronal pyroptosis. Suppression of the STING/ IRE1α signaling pathway can ameliorate NETs-induced neuronal pyroptotic death after TBI.

## Introduction

Traumatic brain injury (TBI) is one of the leading causes of death and disability among all age groups worldwide (1). Following the primary brain injury, the secondary brain injury of TBI is associated with poor neurological outcomes, for which there is no effective treatment (2). TBI-induced neuronal death at the site of injury appears to be the consequence of both the primary brain injury and the secondary brain injury, depending on its localization and temporal course. Neuroinflammation orchestrated by cerebral glia was reported to play a critical role in the pathological process after TBI (3). However, emerging evidence suggested that neuronal cell is also involved in immune responses in brain injury, such as nucleotide-binding oligomerization domain (NOD)-like receptor pyrin domain containing 1 (NLRP1) and NLRP3 inflammasome-mediated inflammation (4, 5). However, the molecular mechanisms responsible for inflammasome-induced neuronal cell death and neuronal inflammation following TBI are complex and still poorly understood.

Pyroptosis, as a novel form of programmed cell death, is characterized by inflammasome-promoted and caspase-1 and/or -11-dependent rapid collapse and gasdermin D (GSDMD)-mediated rupture of cellular integrity, osmotic swelling, and release of pro-inflammatory IL(interleukin)-1β and IL-18 (6). Classically, three types of inflammasomes have currently been validated as playing a major role in the context of neurotrauma (7, 8): NLRP1, NLRP3, and absent in melanoma-2 (AIM2). In the brain, NLRP1 inflammasomes are validated to be primarily expressed in neurons (9). Multiple lines of evidence have confirmed that targeting inhibition of inflammasome priming and activation are critical for ameliorating neuronal cell death and neuroinflammation in secondary brain injury following TBI (10–12). Therefore, targeting NLRP1 inflammation-mediated neuronal pyroptosis may provide new insight and a theoretical basis for developing an effective therapy for TBI.

Since neutrophil extracellular traps (NETs) were first reported by Brinkman in 2004, the NET formation has been reported to be involved in the pathophysiological processes of various brain injuries, such as TBI (13). NETs an extracellular web-like structure released by neutrophils are composed of extracellular double-stranded DNA combined with several components, including histones, myeloperoxidase (MPO), neutrophil elastase, and cathepsin (14). NETs are generated upon excessive neutrophil activation for trapping a broad range of pathogens, including bacteria and viruses, and helping fight infection (15). However, excessive NET formation is generally considered a double-edged sword of innate immunity (16). Excessive NET formation has been recently identified as a novel mechanism contributing to the exacerbation of inflammation (17), the onset of autoimmune disorders (18), and thrombi formation (19). NETs have been reported to be existed in the brain parenchyma (20) and cerebrovascular thrombi (21) of ischemic stroke patients and inhibition of NET formation could improve stroke outcomes in mice subjected to ischemic stroke (20). In addition, previous studies have found that NETs can contribute to brain edema and neurological deficits (22). However, whether NETs are present in the brain tissue of TBI patients, and the biological function and underlying mechanism of NETs in TBI-induced neuronal cell death are still not fully understood.

DNA released by NETs is one of the main sources of circulating free DNA after TBI, and it plays a biological role by activating a series of DNA sensors in the cytosol. Cyclic guanosine 5′-monophosphate (GMP)–adenosine 5′-monophosphate (AMP) synthase (cGAS) is generally considered a crucial cytoplasmic DNA receptor that senses and is stimulated with the combination of endogenous and exogenous double-stranded DNA (dsDNA) (23). cGAS then catalyzes a reaction between guanosine triphosphate (GTP) and adenosine triphosphate (ATP) to produce a second messenger cyclic GMP-AMP (cGAMP), which activates the adaptor protein stimulating Interferon genes (STING) (24). STING is a transmembrane protein located on the endoplasmic reticulum (ER) that functions by regulating innate immunity and sensing pathogens primarily by regulating tank-binding kinase 1 (TBK1)/I interferon regulatory factor 3 (IRF3)/type I interferons (IFNs) pathway (24). Recently studies have revealed that STING not only provokes the TBK1/IRF3/IFNs pathway in a classical way but also is involved in the activation of ER stress and unfolded protein response (UPR) (25–27). STING has been reported to play an important role in regulating the neuroinflammatory process and pyroptosis after TBI (28, 29), but its effects on NLRP1 inflammasome-mediated neuronal cell death have not been explored in TBI. Interestingly, Inositol-requiring enzyme-1 alpha (IRE1α), one of the sensor proteins in the endoplasmic reticulum associated with ER stress, has been shown to regulate NLRP1-mediated neuronal pyroptosis in a neonatal hypoxic-ischemic encephalopathy rat model (30).

In the present study, we hypothesized that NET formation promotes neuronal pyroptosis and neuronal cell death *via* STING/IRE1α pathway after induction of TBI in mice.

## Methods

### Human brain tissues

Human brain tissues were obtained in accordance with an ethically reviewed and approved protocol from Tianjin Medical University General Hospital. Four patients with severe TBI, defined as post-resuscitation Glasgow coma scale (GCS) scores less than 8, were included. Control brain specimens were obtained from three patients with glioma who underwent maximizing resection of malignant glioma to improve survival. None of the patients had any other known neurological disorder. The demographics and clinical characteristics of these patients are shown in **Supplemental Table 1**.

### Quantification of NETs

Plasma was collected from the whole blood of humans by centrifugation at 250 × *g* for 20 min. Plasma DNA was quantified according to the manufacturer’s instructions using the Quant-iT PicoGreen dsDNA Assay kit (P11496, Invitrogen). We also developed a capture ELISA based on citrullinated histone H3 associated with DNA (H3cit-DNA complex), as reported previously (31). For the capture of antibodies, 5μg/ml anti-H3Cit (ab5103, Abcam, UK) was coated onto 96-well plates overnight at 4°C. The plates were then blocked with 5% BSA for 2 h at room temperature. After being washed with washing buffer (300 μL each), 50 μL of plasma was added into the wells with 80 μL incubation buffer containing a peroxidase-labeled anti-DNA mAb (Cell Death ELISA, 11774425001, Roche, Basel, Switzerland). The plates were then incubated at room temperature for 2 h. After being washed six times, the plate was developed with 100 μL ABST substrate. The absorbance (OD) at 450 nm was measured after 30 min incubation in the dark.

### Animals

Male C57BL/6 mice (8-10 weeks old and 25-30g), were purchased from the Experimental Animal Laboratories of the Academy of Military Medical Sciences (Beijing, China). All mice were housed in a temperature (20°C–24°C) and humidity (50%–60%) controlled environment with food and water available *ad libitum* under a standard 12-h light/dark cycle. All experimental procedures were conducted in strict accordance with the National Institutes of Health Guide for the Care and Use of Laboratory Animals and approved by Tianjin Medical University Animal Care and Use committee.

### Experimental design

All experimental mice were randomly assigned to five experimental groups. Surgeries, histological, and neurological function assessments were carried out in a blinded manner.

#### Experiment 1

To examine the role of neutrophils in secondary neuronal cell death following TBI, 36 mice were randomly assigned into the following three groups (n=12/group): sham, TBI + Isotype, and TBI + Anti-Ly6G for western blot analysis and histological analysis. To assess the role of neutrophils in neurological function after TBI, a total of 36 mice were randomly divided into three groups (n=12/group): sham, TBI + Isotype, and TBI + Anti-Ly6G. On days 1, 3, 5, and 7post-injuries, the rotarod test and modified neurological severity score (mNSS) were conducted to assess post-trauma motor functions.

#### Experiment 2

To investigate the effects of neutrophil extracellular trap and Stimulators of interferon genes (STING) agonist in secondary neuronal cell death following TBI, 48 mice were randomly assigned into the following four groups (n=12/group): sham, TBI + vehicle, TBI + DNase 1, and TBI + DNase 1 + cGAMP for western blot analysis and histological analysis.

#### Experiment 3

To investigate the effects of neutrophil extracellular trap formation inhibitor and STING agonist in secondary neuronal cell death following TBI, 48 mice were randomly assigned into the following four groups (n=12/group): sham, TBI + vehicle, TBI + Cl-amidine, and TBI + Cl-amidine + cGAMP for western blot analysis and histological analysis. To assess the effects of neutrophil extracellular trap formation inhibitor and STING agonist in neurological function after TBI, a total of 48 mice were randomly divided into four groups (n=12/group): sham, TBI + Isotype, and TBI + Anti-Ly6G. On days 1, 3, 5, and 7 post-injuries, the rotarod test and mNSS were conducted to assess post-trauma motor functions.

#### Experiment 4

To determine the effects of neutrophil extracellular trap formation and STING inhibitor in secondary neuronal cell death following TBI, 48 mice were randomly assigned into the following four groups (n=12/group): sham, TBI + Ad-con, TBI +Ad-PAD, and TBI + Ad-PAD + C-176 for western blot analysis and histological analysis.

#### Experiment 5

To further explore the IRE1α signaling pathway in neutrophil extracellular traps-mediated neuronal pyroptosis after ICH, a total of 24 mice were randomized into four groups (n=6/group): sham, TBI + Ad-con, TBI +Ad-PAD, and TBI + Ad-PAD + kira6 for western blot analysis. To assess the IRE1α signaling pathway in neurological function after TBI, 48 mice were randomly divided into four groups (n=12/group): sham, TBI + Ad-con, TBI +Ad-PAD, and TBI + Ad-PAD + kira6. On days 1, 3, 5, and 7 post-injuries, the rotarod test and mNSS were conducted to assess post-trauma motor functions.

### TBI model

TBI was induced by a digital electromagnetically controlled cortical impact (CCI) device (CCI-6.3 device, Custom Design & Fabrication, USA) as previously described (32). Briefly, each mouse was anesthetized with 1–1.5% isoflurane in 30% oxygen and 70% nitrous oxide during the surgical procedure, and the body temperature was maintained at 37 ± 0.5°C using a heating blanket. After fixation on a custom head-fixing apparatus, a craniotomy was performed using a high-speed micro drill on the right parietal cortex (2.5 mm posterior from the bregma and 2.5 mm lateral to the sagittal suture) (33). A 4-mm-flat impactor tip was used to conduct a unilateral 2.2 mm depth impact on the mice at 5 m/s with a 200 ms dwell time. The sham mice were subjected to the same surgical procedure without the infliction of CCI. All procedures were performed with a strict aseptic technique.

### Injection of adenoviruses

Recombinant PAD4 adenovirus (Adeno-PAD4; Adeno-CMV-Padi4-3*flag-tagged SV40-EGFP, 4 × 10^10^ plaque-forming-unit/mL) and empty adenovirus (Adeno-CMV-3*flag-tagged SV40-EGFP) were purchased from Genechem (Shanghai, China). A total of 4 μL of Adeno-PAD4 (Ad-PAD4) or Adeno-EGFP (Ad-con) was injected stereotactically into two sites of the right cortex (1.5 mm caudal and 1.5 mm rostral from lesion epicenter) at a dose of 2 μL, respectively, 24 h before mice subjected to CCI. The injections were performed with a 5 μL Hamilton syringe (Hamilton Company, USA) at a flow rate of 0.5 μL/min through the hole at a depth of 1.0 mm (33, 34). After the injection, the needle was held for 10 min before retraction, and the scalp was stitched.

### Neutrophil depletion

As previously described (35), to deplete neutrophils *in vivo*, each mouse received an intraperitoneal (i.p.) injection of 100 μg monoclonal anti-mouse Ly6G antibody (1A8 clone; specific for neutrophils, BE0075-1, BioXCell, NH) 24 h before TBI and 24 h after TBI. Each control mouse received 100 μg of the isotype antibody rat IgG2a in the same manner.

### Drug administration

#### Human recombinant DNase 1 treatment

As previously described (13), mice were intravenously (i.v.) injected with 5 mg/kg rh DNase 1 (Deoxyribonuclease 1 human recombinant; enz-319-10,000 IU, ProSpec-Tany TechnoGene Ltd., Rehovot, Israel) or vehicle (8.77 mg/mL sodium chloride and 0.15 mg/mL calcium chloride) 1 h after TBI and then injected daily until the mice were euthanized.

#### Peptidyl arginine deiminases inhibitor treatment

A stock solution of the PAD inhibitor, Cl-amidine (GC35706, GLPBIO, USA), was diluted in saline (5% v/v). Then, as previously mentioned (13), mice were assigned to the injection intraperitoneally of Cl-amidine at a dose of 10 mg/kg or an equal volume of vehicle (saline containing 5% DMSO) 10 min after TBI and once per day for three consecutive days.

#### Stimulators of interferon genes antagonist administration

The STING antagonist C-176 (S6575, Selleck, USA) was dissolved in a stock solution containing 5% dimethyl sulfoxide (DMSO) and 95% corn oil and then diluted in saline (5% v/v). As previously reported (29), 10 mg/kg C-176 or an equal volume of vehicle (saline containing 5% stock solution) was administered i.p. 1 h after TBI and once per day for three consecutive days.

#### 2’3’-cGAMP treatment

As previously reported (36, 37), 1 mg/kg 2′3′-cGAMP (tlr-lnacga23-1; *In vivo*Gen, San Diego, USA) or vehicle (phosphate-buffered saline, PBS) was administered intravenously 10 min before TBI and once per day for three consecutive days.

#### Inositol-requiring enzyme 1α inhibitor administration

The IRE1α inhibitor kira6 (S8658, Selleck, USA) was dissolved in a stock solution containing 5%DMSO, 30% Tween-80, and 65% corn oil and then diluted in saline (5% v/v). As previously reported (38), 10 mg/kg kira6 or an equal volume of vehicle (saline containing 5% stock solution) was administered i.p. 1 h after TBI and once per day for three consecutive days.

### Neurological assessment

Neurological function was evaluated at 1, 3, 5, and 7 d after TBI using the well-established mNSS, as described previously (39). The mNSS consists of a motor (muscle state and abnormal action), sensory (visual, tactile), reflex, and balance tests. The higher score represents the more serious neurological impairment (normal score, 0; maximal score, 18).

### Rotarod test

As described previously (40), the rotarod test was performed to assess the sensorimotor function, coordination, and balance at 1, 3, 5, and 7 d post-injury with an accelerating Rota-rod apparatus (RWD Life Science, Shenzhen, China). Before induction of CCI, mice in each group were trained for three consecutive days. Each mouse was placed in each lane on the rotating cylinder, which accelerated from 4 to 40 rpm/min within 5 min. The latency to fall for each mouse was recorded. Each mouse was tested three times with an interval of 30 min between trials, and the average latency to falling was used for analysis.

### H&E staining

H&E staining was performed to evaluate damaged cells as previously described (41). The paraffin-embedded 4% paraformaldehyde-fixed brains were sliced into 10um thick sections. The brain sections were then deparaffinized with xylene and dehydrated in a graded series of alcohols. Sections were incubated with hematoxylin (Sigma Aldrich) for 3 mins and washed three times with PBS. Then, sections were stained with eosin for 4 min. The average number of normal neurons and damaged neurons were calculated by ImageJ software (Version 1.46r, Wayne Raband, USA).

### Nissl staining

Nissl staining was performed to evaluate neuronal damage as previously described (32). The paraffin-embedded 4% paraformaldehyde-fixed brains were sliced into 10um thick sections. The brain sections were then deparaffinized with xylene and dehydrated in a graded series of alcohols. Sections were incubated with Nissl staining solution (Sigma Aldrich) for 20 mins at 60°C in the oven. The damaged neurons were characterized by a shrunken cytoplasm and condensed staining, while normal neurons were characterized by a large and full soma. The average number of normal neurons and damaged neurons were calculated by ImageJ software (Version 1.46r, Wayne Raband, USA).

### Immunofluorescence staining

After the sacrifice of mice, the brains were removed and immersed in 4% paraformaldehyde, 15% sucrose, and 30% sucrose to complete fixation and dehydration. Brains were then sliced into 8μm-thick coronal sections using a cryostat (Leica, Model CM1950, Germany). After being washed in PBS, the sections were permeabilized with 0.1% Triton X-100 (Sigma Aldrich) for 30 mins and incubated with 3% BSA for 1h at room temperature. Thereafter, sections were incubated overnight at 4°C with primary antibodies, including mouse anti-NeuN (1:1000, ab104224), rabbit anti-NeuN (1:1000, ab177487), rabbit anti-H3Cit (1:1000, ab5103, all from Abcam, UK), rabbit anti-STING (1:500, 13647, Cell Signaling Technology), goat anti-MPO (1:500, AF3667, R&D Systems), goat anti-CD31(1:200, AF3628, R&D Systems), rat anti-mouse Ly6G (1:200, 551459, BD Biosciences), mouse anti-Caspase1 (1:500, sc-56036, Santa Cruz Biotechnology), mouse anti-NLRP1 (1:500, sc-390133, Santa Cruz Biotechnology). The sections were then incubated with the species-appropriate Alexa Fluor-conjugated IgG (1:500, Invitrogen, USA) for 1h at room temperature. DNA was stained with 4’,6-diamidino-2-phenylidole (DAPI, Abcam). Staining was visualized and captured by an inverted fluorescence microscope (Olympus, Japan). Images were then analyzed using ImageJ software (Version 1.46r, Wayne Raband, USA).

### Terminal deoxynucleotidyl transferase dUTP nick-end labeling assay

For quantification of neuronal apoptosis at 3 days after TBI, double staining of neuron marker NeuN (red) and TUNEL (green) was conducted using the *In Situ* Cell Death Detection kit (Roche, South San Francisco, CA, USA), according to the manufacturer’s instructions. Coronal sections were counterstained with mouse anti-NeuN (1:1000, ab104224, Abcam, UK), at 4°C overnight and subsequently incubated with the *In Situ* Cell Death Detection kit and a secondary donkey anti-mouse Alexa 594 antibody for 1h at 37°C in the dark. The average number of TUNEL-positive neurons in the contused cortex was calculated by ImageJ software (Version 1.46r, Wayne Raband, USA).

### Western blot analysis

Total proteins in the brain tissues of TBI mice were extracted using RIPA lysis buffer (Sigma-Aldrich, MO, USA). Proteins were separated by sodium dodecyl sulfate-polyacrylamide gel electrophoresis (SDS-PAGE) and transferred to a 0.45um pore size polyvinylidene difluoride (PVDF) membranes (Millipore, Temecula, CA, USA). The membranes were blocked with 5% skimmed milk for 2 h and then incubated primary antibodies: including rabbit anti-IREα (1:1000, 3294), rabbit anti-ASC (1:1000, 67824), rabbit anti-Histone H3 (anti-H3; 1:1000, 9715), rabbit anti-STING (1:1000, 13647), rabbit anti-β-actin (1:2000, 4970, all from Cell Signaling Technology, MA), rabbit anti-H3Cit (1:1000, ab5103), rabbit anti-p-IREα (1:1000, ab124945), rabbit anti-IL-18 (1:1000, ab207323), rabbit anti-MPO (1:1000, ab208670), rabbit anti-PAD4 (1:1000, ab96758, all from Abcam, UK), anti-Caspase1 (22915–1-AP, 1:2000, Proteintech Group, Wuhan, China), anti-GSDMD (AF4012, 1:2000, Affinity Biosciences), mouse anti-NLRP1 (1:500, sc-390133, Santa Cruz Biotechnology). The membranes were then washed three times and incubated with species-appropriate horseradish peroxidase (HRP)-conjugated secondary antibodies (1:5000, Cell Signaling Technology, USA) for 1h at room temperature. Finally, the immunoblot bands were visualized using ECL under an imaging system (Bio-Rad, Hercules, CA, USA). Protein quantification was measured in optical density units using Image Lab-5.2.1 software (Bio-Rad, CA, USA) and was normalized to the corresponding sample expression of β-actin.

### Statistical analysis

All statistical analysis was performed with Graph-Pad Prism software (Graph Pad Software, Version8.1.2 San Diego, CA, USA). Multiple comparisons were analyzed by one-way analysis of variance (ANOVA) followed by Tukey’s multiple comparison *post hoc* test. The results were expressed as means ± (SD). Comparison between 2 groups was performed by unpaired Student’s t-test or Mann-Whitney test. A value of P < 0.05 was considered statistically significant.

## Results

### NETs formation correlates with elevated ICP and worse neurological function in TBI patients

**Figure 1:**
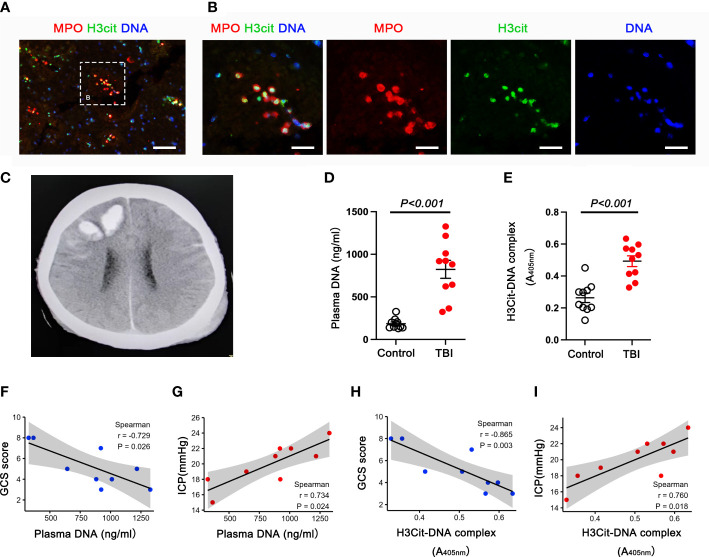
NETs are found in the brain and circulating blood from TBI patients and have a significant correlation with GCS and ICP. **(A, B)** Representative immunofluorescence images **(A)** displaying the NET formation (H3Cit-positive neutrophils) in the brain tissue sections from TBI patients. Enlarged images on the right side **(B)**. Scale bar = 50 μm **(A)**; Scale bar = 200 μm **(B)**. A representative CT image from a TBI patient **(C)**. The image shows the right frontal contusion and hematoma. **(D, E)** Quantification of the Levels of plasma DNA **(D)**, plasma H3Cit-DNA complex **(E)** in the peripheral blood from healthy donors (n = 10) and TBI patients (n = 10). **(F-I)** Correlation among the levels of plasma DNA, plasma H3Cit-DNA complex with Glasgow Coma Scale (GCS) and intracranial pressure (ICP). Spearman correlation analysis was applied with r=-0.729, P =0.026 **(F)** (plasma DNA correlate with GCS score), r=0.734, P =0.024 **(G)** (plasma DNA correlate with ICP), r=-0.865, P =0.003 **(H)** (plasma H3Cit-DNA complex correlate with GCS score), r=0.734, P =0.024 **(I)** (plasma H3Cit-DNA complex correlate with ICP). The statistical graph shows that the higher the level of NETs, the greater the degree of TBI.

To explore whether neutrophils are present in the brain tissues of TBI patients, we obtained brain specimens from four severe TBI patients who underwent a decompressive craniotomy to remove the hematoma and/or to reduce life-threatening high intracranial pressure. We found that infiltration of both neutrophils and neutrophils-derived NETs was significantly increased in the brain tissues of TBI patients (**Figures 1A, B**). A typical head CT image of a patient with TBI observed a brain contusion in the right frontal lobe with intracranial hematoma (**Figure 1C**). Plasma cell-free DNA (cf-DNA) (**Figure 1D**), a rough biomarker of NETs, and plasma H3Cit-DNA complex (**Figure 1E**), a specific biomarker of NETs, were significantly elevated in TBI patients compared with matched healthy donors. Notably, a significant inverse correlation was observed between plasma DNA levels and GCS score, and a significant positive correlation was detected between plasma DNA levels and ICP (**Figures 1F, G**). Similarly, plasma H3Cit-DNA complex levels positively correlated with ICP, whereas inversely correlated with the GCS score. (**Figures 1H, I**). These results raise the unexplored possibility that NETs contribute to neurological deficits and cerebral edema after TBI.

### Neutrophil infiltration and NETs formation in brain tissues are around the trauma area in mice with TBI

**Figure 2:**
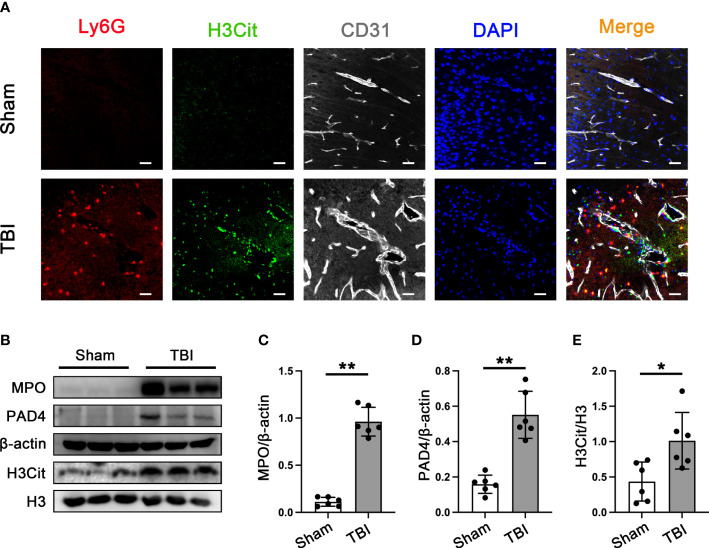
Neutrophils infiltration and NETs formation in mice brain tissue. **(A)** Representative immunofluorescence staining of Ly6G-positive neutrophils (red) and H3cit -positive(green) and CD31-labeled blood vessels (white) in the mice brain tissue sections post-CCT 3 days. Nuclei were stained with DAPI (blue). **(B)** Representative Western blot bands of MPO, PAD4, H3Cit, and H3 and statistical analysis expression level of MPO **(C)**, PAD4 **(D)**, and H3Cit **(E)** in mice brain tissue (n=6). Data are represented as mean ± SD. *P < 0.05, **P < 0.01.

To verify the presence of neutrophil infiltration and NETs formation in the brain tissue of 3d mice after TBI. Double immunofluorescence staining of Ly6G and NETs-specific markers (citrullinated histone H3, H3cit), neutrophil, and neutrophil-derived NETs could be simply observed in the mouse brain sections after TBI (**Figure 2A**). It has been confirmed that the peak of neutrophil infiltration in mouse brain tissue is 3 days after TBI (42). Western Blot results that the protein expression of MPO, PAD4, and H3Cit was significantly increased in the cortex around the trauma area 3d post-TBI (**Figures 2B–E**).

### Neutrophil depletion ameliorates neuronal function and neuronal death induced by NLRP1 after TBI

**Figure 3:**
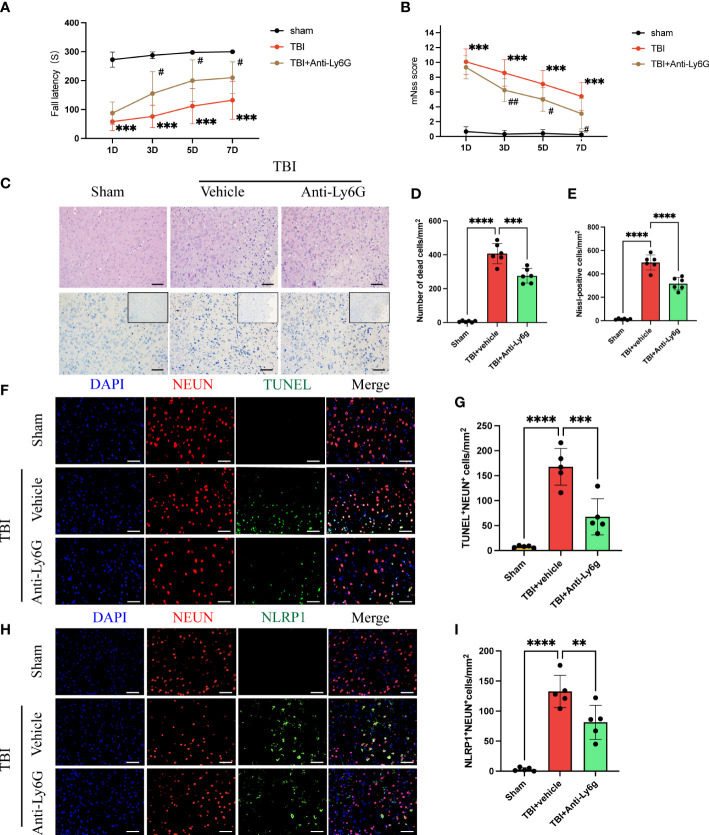
Neutrophil depletion ameliorates neurological function and reduces NLRP1-induced neuronal death after TBI. Rotary test **(A)** and modified neural severity scores (mNSS, **B**) were used to evaluate the neural function of mice treated with vehicle and Anti-Ly6G compared with the Sham group at 1 d, 3 d, 5 d, 7 d after TBI (n = 12). **(C)** Hematoxylin and eosin staining and Nissl staining of the cortex around the traumatic area 3 days after TBI(n=6). Count the number of dead cells of Hematoxylin and eosin staining **(D)** and Nissl staining **(E)**. Scale bar = 100 μm. **(F)** Representative immunofluorescence images of TUNEL-positive(green) cells and NEUN-positive (red) neurons in the peritraumatic cortex (n=6). Count and compare the number of TUNEL-positive neurons in different groups **(G)**. **(H)** Representative immunofluorescence images of NLRP1-positive(green) cells and NEUN-positive (red) neurons. Count and compare the number of NLRP1-positive neurons in different groups **(I)**. Nuclei were stained with DAPI (blue). Scale bar = 100 μm. Data are represented as mean ± SD. **P < 0.01, ***P < 0.001 and ****P < 0.0001. compared within two groups. #p < 0.05, ##p < 0.01 and vs vehicle group.

The rotary test and mNSS were used to assess neurological function in the acute phase after TBI. we found that anti-Ly6G treatment effectively improved neurological deficits in mice at 3 d after TBI (**Figures 3A, B**). Hematoxylin and eosin staining and Nissl staining showed massive degeneration and necrosis of neuronal cells 3 days after TBI. However, the number of neuronal deaths was significantly reduced in anti-Ly6G-treated mice compared with the vehicle group (**Figures 3C–E**). Additionally, we observed neuronal death by immunofluorescence staining of NEUN (red) and TUNEL (green) in the different groups (**Figures 3F, G**). The results showed that neuronal death was significantly reduced in the anti-Ly6G treatment group compared with the TBI+ vehicle group. NLRP1 (nucleotide-binding domain leucine-rich repeat pyrin domain containing 1) inflammasomes are primarily expressed in neurons and is closely related to neuronal pyroptosis. The results observed that the number of NLRP1 positive neurons in the anti-Ly6G treatment group was significantly reduced compared with the vehicle group (**Figures 3H, I**). Therefore, neutrophil infiltration was closely related to NLRP1-mediated neuronal pyroptosis after TBI by double immunofluorescence staining of NEUN (red) and NLRP1(green).

### Degradation of NETs-associated DNA ameliorates neuronal pyroptosis by inhibiting the STING- IRE1α-NLRP1 pathway after TBI

**Figure 4:**
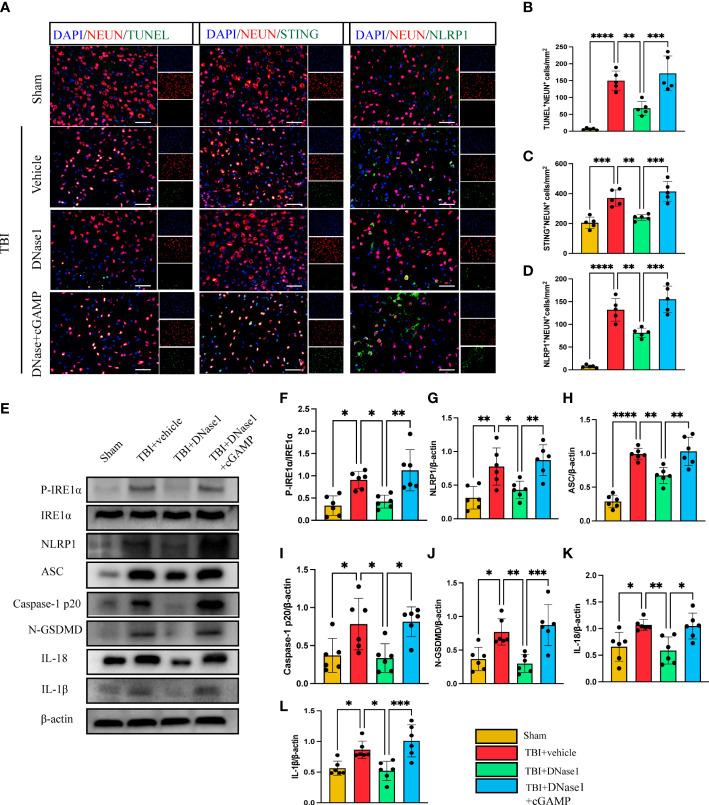
DNase 1 ameliorates neurons’ pyroptosis by inhibiting the STING-IRE1α-NLRP1 pathway after TBI. **(A)** Representative fluorescent double stain images of NEUN-positive neurons with TUNEL-positive cells (green), STING-positive cells (green) and NLRP1-positive cells (green) in the peritraumatic cortex(n=5). Nuclei were stained with DAPI (blue). Scale bar = 50 μm. Count and analyze the number of double-stained positive cells of NEUN-positive neurons with TUNEL-positive cells **(B)**, STING-positive cells **(C)** and NLRP1-positive cells **(D)**. **(E)** Representative Western blot bands of P-IRE1α, NLRP1, ASC, Caspase-1 p20, N-GSDMD, IL-18, IL-1βand statistical analysis expression level of P-IRE1α **(F)**, NLRP1 **(G)**, ASC **(H)**, Caspase-1 p20 **(I)**, N-GSDMD **(J)**, IL-18 **(K)**, IL-1β **(L)** in brain tissue of mice post-TBI 3 days (n=6). Data are presented as mean ± SD. *P < 0.05, **P < 0.01, ***P < 0.001 and ****P < 0.0001.

We next confirm whether the degradation of NET-associated DNA with DNase 1 could ameliorate neuronal pyroptosis by inhibiting the STING-IRE1α-NLRP1 pathway. Immunofluorescence staining showed the number of TUNEL-positive neurons remarkably increased in the cortex around the trauma area of TBI mice compared with the sham group (**Figures 4A, B**). In TBI mice treated with Dnase 1 the number of TUNEL-positive neurons significantly reduced in the peripheral cortex of the trauma area (**Figures 4A, B**). Phosphorylated STING is an activated form of STING. In order to verify the activation of STING in neurons after TBI, immunofluorescence staining was used to observe the expression of P-STING in neurons (**Supplementary Figure 1**). The results showed that the expression of P-STING increased significantly in neurons after TBI, and DNase1 treatment could reverse this result. This effect of DNase 1 was abolished in mice that also received the second messenger cGAMP 3 d after TBI (**Figures 4A, B**). Consistent with this result, compared with TBI +vehicle group, DNase 1 treatment could significantly reduce the number of STING-positive neurons. However, cGAMP treatment can reverse the treatment advantage of DNase 1 (**Figures 4A, C**). Meanwhile, the number of NLRP1-positive neurons was significantly reduced in DNase 1 treatment mice with TBI. Interestingly, cGAMP treatment also reversed the therapeutic effects of DNase 1 (**Figures 4A, D**). Therefore, STING can activate the pyroptosis pathway of neurons. We subsequently found that the protein expression of P- IRE1α, NLRP1, ASC, Caspase-1 p20, N-GSDMD, IL-18, and IL-1β were remarkably upregulated in the cortex of mice subjected to TBI (**Figures 4E–L**). DNase 1 treatment significantly rescued the activation of the STING-IRE1α-NLRP1 pathway and reduced protein products associated with neuronal pyroptosis. However, treatment with the cGAMP effectively reversed the effect of DNase 1 on the STING-IRE1α-NLRP1 pathway (**Figures 4E–L**). These results showed that degradation of neutrophil-released NETs associated DNA with DNase 1 could recuse neuronal pyroptosis by inhibiting the STING-IRE1α-NLRP1 pathway.

### Inhibition of PAD4 ameliorates neurological function and neuronal pyroptosis by inhibiting the STING/IRE1α-NLRP1 pathway after TBI

**Figure 5:**
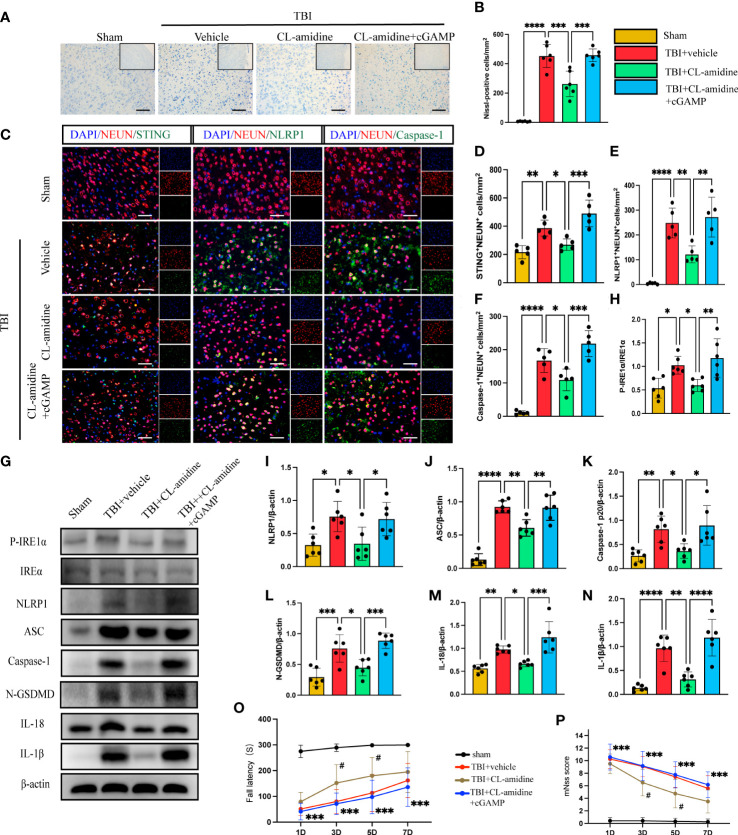
Inhibition of PAD4 ameliorates neurons’ pyroptosis by inhibiting the STING-IRE1α-NLRP1 pathway. **(A)** Nissl staining images of the peri-injured brain tissue of mice. Count the number of Nissl-positive cells in the different groups **(B)**. Scale bar = 100 μm. **(C)** Representative fluorescent double stain images of NEUN-positive neurons with STING-positive cells (green), NLRP1-positive cells (green) and Caspase-1-positive cells (green)in the peritraumatic cortex(n=5). Nuclei were stained with DAPI (blue). Scale bar = 50 μm. Count and analyze the number of double-stained positive cells of NEUN-positive neurons with STING-positive cells **(D)**, NLRP1-positive cells **(E)** and Caspase-1-positive cells **(F)** in the different groups. **(G)** Representative Western blot bands of P-IRE1α, NLRP1, ASC, Caspase-1 p20, N-GSDMD, IL-18, IL-1βand statistical analysis expression level of P-IRE1α **(H)**, NLRP1 **(I)**, ASC **(J)**, Caspase-1 p20 **(K)**, N-GSDMD **(L)**, IL-18 **(M)**, IL-1β **(N)** in brain tissue of mice post-TBI 3 days (n=6). Rotary test **(O)** and mNSS **(P)** were used to evaluate the neural function of mice treated with PBS and Anti-Ly6G compared with the Sham group at 1 d, 3 d, 5 d, 7d after TBI (n = 12). Scale bar = 50 μm. Data are presented as mean ± SD. *P < 0.05, **P < 0.01, ***P < 0.001 and ****P < 0.0001 compared within two groups. ^#^p < 0.05 and vs CL-amidine+ cGAMP group.

Peptidylarginine deiminase 4 (PAD4) catalyzes histone citrullination that plays a key role in NETs formation (43, 44). We examined a hypothesis on whether inhibition of PAD4(CL-amidine) to reduce NETs-formation improved neural function and reduced neuronal pyroptosis. First, Nissl staining showed that the number of neuronal deaths was significantly reduced in CL-amidine treatment mice compared with the vehicle group (**Figures 5A, B**). However, treatment with cGAMP could revere the effectiveness of CL-amidine after TBI (**Figures 5A, B**). First, confocal microscopy was used to observe the brain tissue stained by immunofluorescence, and it was found that STING/NLRP1 and STING/Caspase-1 could be expressed simultaneously in the same neuron (**Supplementary Figure 2**). In addition, immunofluorescence staining showed that treatment with Cl-amidine significantly reduced the number of STING-positive, NLRP1-positive, and Caspase-1-positive neurons in the cortex surrounding the trauma area (**Figures 5C–F**). On the contrary, cGAMP treatment could block the effectiveness of CL-amidine (**Figures 5C–F**). The protein levels of P- IRE1α, NLRP1, ASC, Caspase-1 p20, N-GSDMD, IL-18, and IL-1β were detected by western blot and it was found that the protein expression level of STING/P- IRE1α-NLRP1 pathway and neuronal pyroptosis-related protein products in the CL-amidine treatment group were significantly down-regulated compared with TBI+ vehicle group (**Figures 5G–N**). However, the protein level of the STING-IRE1α-NLRP1 pathway and neuronal pyroptosis-related protein products were still up-regulated in the mice that interfered with both CL-amidine and cGAMP (**Figures 5G–N**). Meanwhile, the results of the rotary test and mNSS scores showed that Inhibition of PAD4 could remarkably ameliorate neurological deficits of mice on day 3 after TBI (**Figures 5O, P**). These results showed that Inhibition of PAD4 could ameliorate neurological deficits and recuse neuronal pyroptosis by inhibiting the STING-IRE1α-NLRP1 pathway.

### Activated PAD4 promotes neuronal pyroptosis through the STING-IRE1α-NLRP1 pathway after TBI

**Figure 6:**
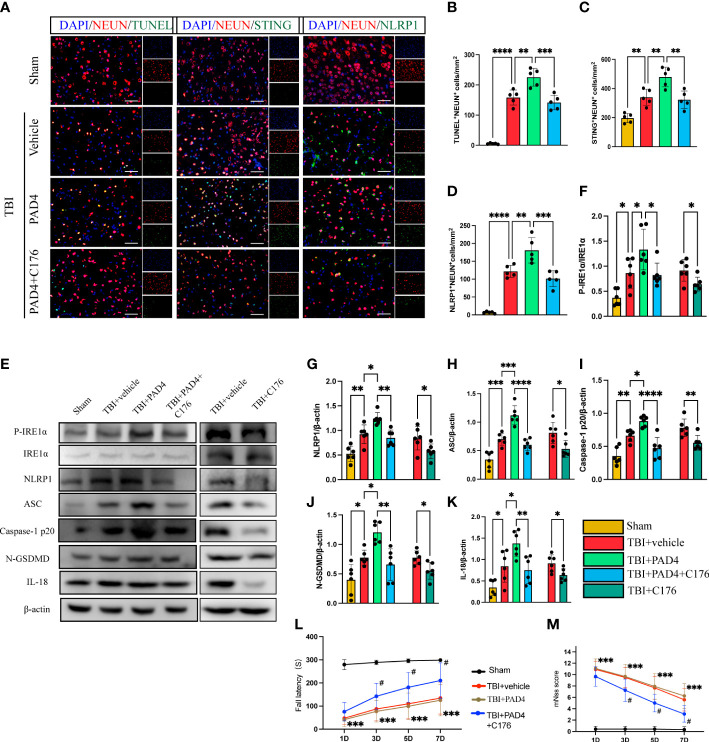
Overexpression of PAD4 exacerbates neurons’ pyroptosis by promoting the STING-IRE1α-NLRP1 pathway. **(A)** Representative fluorescent double stain images of NEUN-positive neurons with TUNEL-positive cells (green), STING-positive cells (green) and NLRP1-positive cells (green) in the peritraumatic cortex(n=5). Nuclei were stained with DAPI (blue). Scale bar = 50 μm. Count and analyze the number of double-stained positive cells of NEUN-positive neurons with TUNEL-positive cells **(B)**, STING-positive cells **(C)** and NLRP1-positive cells **(D)**. **(E)** Representative Western blot bands of P-IRE1α, NLRP1, ASC, Caspase-1 p20, N-GSDMD, IL-18 and statistical analysis expression level of P-IRE1α **(F)**, NLRP1 **(G)**, ASC **(H)**, Caspase-1 p20 **(I)**, N-GSDMD **(J)** and IL-18 **(K)** in brain tissue of mice post-TBI 3 days (n=6). Rotary test **(L)** and mNSS **(M)** were used to evaluate the neural function of mice treated with PBS and Anti-Ly6G compared with the Sham group at 1 d, 3 d, 5 d, 7d after TBI (n = 12). Data are presented as mean ± SD. *P < 0.05, **P < 0.01, ***P < 0.001 and ****P < 0.0001 compared within two groups. ^#^p < 0.05 and vs PAD4+C176 group.

To study whether that increased formation of NET, orchestrated by PAD4, contributed to neuronal pyroptosis, we first studied the role of overexpression of PAD4 on neuronal pyroptosis in mice with TBI. Immunofluorescence staining showed extensive expression of recombinant adeno-PAD4-EGFP-infected cells in the cortex 3 d after injection (**Supplementary Figure 3**). It can be seen from the figure that the expression of PAD4 is mainly concentrated in neutrophils. Immunofluorescence staining showed that administration of PAD4 adenovirus into the cortex led to a remarkable increase in the number of neuronal death (TUNEL-positive neurons), STING-positive neurons, and NLRP1-positive neurons (**Figures 6A–D**). Interestingly, STING inhibitor C176 could block the function of PAD4 adenovirus (**Figures 6A–D**). Meanwhile, the protein expression level of the STING- P- IRE1α-NLRP1 pathway and neuronal pyroptosis-related protein products were also detected by Western Blot. The results indicated that the protein levels of P- IRE1α, NLRP1, ASC, Caspase-1 p20, N-GSDMD, and IL-18 were significantly increased after the administration of PAD4 adenovirus into the cortex (**Figures 6E–K**). However, STING inhibitor C-176 could decrease the expression level of related proteins after TBI and reverse the effect of PAD4 adenovirus (**Figures 6E–K**). Finally, we evaluated neurological function during the acute phase post-TBI by rotary test and mNSS scores after administration of PAD4 adenovirus and PAD4 adenovirus + C176. The results showed that the PAD4 adenovirus group aggravated nerve damage after TBI, and C176 improved the adverse effects caused by PAD4 adenovirus (**Figures 6L, M**). These results showed PAD4 played a key role in NLPR1-induced neuronal pyroptosis *via* STING/IRE1α pathway.

### Inhibition of IRE1α improves through the STING-IRE1α-NLRP1 pathway after TBI

**Figure 7:**
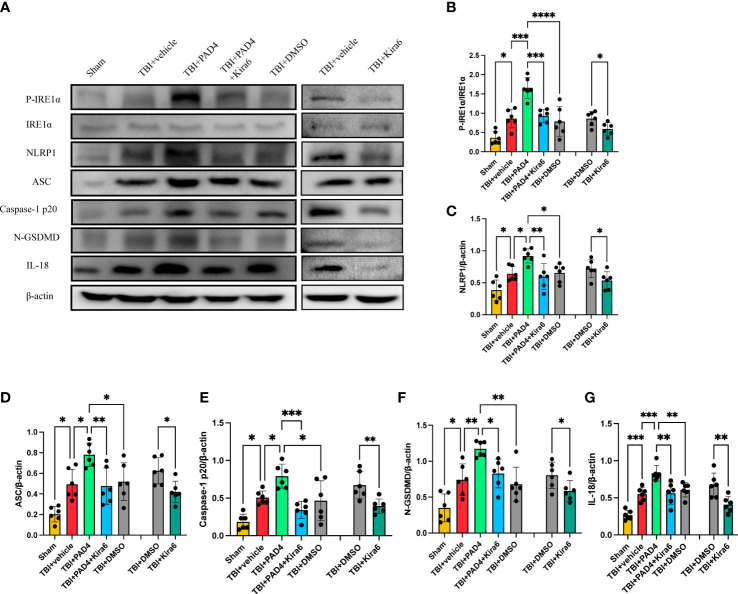
Inhibition of IRE1α ameliorates neurons’ pyroptosis by inhibiting the STING-IRE1α-NLRP1 pathway. **(A)** Representative Western blot bands of P-IRE1a **(B)**, NLRP1 **(C)**, ASC **(D)**, Caspase-1 p20 **(E)**, NGSDMD **(F)** and IL-18 **(G)**. and statistical analysis expression level of STING **(B)**, P-IRE1α **(B)**, NLRP1 **(C)**, ASC **(D)**, Caspase-1 p20 **(E)**, N-GSDMD **(F)** and IL-18 **(G)** in brain tissue of mice post-TBI 3 days (n=6). Data are presented as mean ± SD. *P < 0.05, **P < 0.01, ***P < 0.001 and ****P < 0.0001 compared within two groups.

To explore whether IRE1α is a key part of the pathway that NETs cause neuronal pyroptosis. We used IRE inhibitor Kira6 after the administration of PAD4 adenovirus into the cortex to observe the expression of pathway proteins. The results found that the protein expression of P-IRE1α was significantly increased in the PAD4 adenovirus group and IRE inhibitor Kira6 could block the effect of PAD4 adenovirus (**Figures 7A, B**). Meanwhile, Immunoblotting showed the protein expression of NLRP1, ASC, Caspase-1 p20, N-GSDMD, and IL-18 were significantly reduced after the administration of Kira6 compared with the DMSO group and the PAD4 adenovirus group. (**Figures 7A, C–G**). These data suggest that IRE1α plays a key role in NLRP1-induced neuronal pyroptosis by promoting NETs formation.

## Discussion

In the present study, we first confirmed the increased infiltration of NETs in the contused brain of TBI patients. Furthermore, we found that increased markers of NET formation in the circulation plasma of TBI patients correlate with elevated intracranial pressure (ICP) and worse neurological function. Moreover, we found that neutrophils and NET formation in the cortex parenchyma is increased in mice subjected to TBI, and depletion of neutrophils with Anti-Ly6G effectively reduced NET formation and ameliorated neurological deficits and attenuated NLRP1-mediated neuronal cell pyroptosis. In addition, we demonstrated that the degradation of NETs or inhibition of NET formation attenuated NLRP1-mediated neuronal cell pyroptosis in a STING-dependent way. Notably, after overexpression of NET formation by adenoviruses in TBI mice, the NLRP1-mediated neuronal cell pyroptosis increased, whereas this effect was effectively abolished by a STING inhibitor. Another novel finding of the current study is that NETs can activate STING-dependent ER stress and IRE1α inhibitor effectively attenuates NETs-induced neuronal pyroptotic death after TBI. Collectively, NET formation promotes NLRP1 inflammasome-mediated neuronal pyroptosis after TBI, at least partly through STING/IRE1α signaling pathway.

Pathologically, programmed neuronal cell death and secondary inflammation are two common hallmarks of TBI (45). Programmed neuronal cell death is characterized by any form of cell death caused by either an intracellular or extracellular death program, including apoptosis, necroptosis, autophagy, and pyroptosis (45). Intriguingly, unlike any other programmed cell death, pyroptosis is a highly inflammatory form of programmed cell death mediated by cleaved caspase-1 and/or caspase-11 (46). Previous studies demonstrated that NLRP1 inflammasomes are primarily expressed in neurons (9), and NLRP1 inflammasomes modulate caspase-1 cleavage and consequent IL-1β and IL-18 processing (47). In a rat model of spinal cord injury, the mRNA and protein of NLRP1 were significantly elevated from 18 to 24 hours after spinal cord injury (48). A previous study showed that silencing NLRP1 inflammasome reduced neuronal cell pyroptosis in a mouse model of Alzheimer’s disease (49). Importantly, emerging evidence also demonstrated that NLRP1 inflammasome activation promotes neuronal cell pyroptosis and neurological deficits after brain injury (5, 30, 47, 50). Similarly, we observed that the expression of NLRP1 was significantly upregulated and inhibition of the STING/IRE1α signaling pathway effectively rescued NLRP1-dependent neuronal pyroptosis and secondary neuronal apoptosis in mice subjected to TBI.

NETs are lattices of extracellular DNA, released by neutrophils meant to trap and kill pathogens. However, there is increasing evidence to suggest that dysfunction of the NET inhibition mechanism and excessive NET production are also important pathogenic mechanisms that may lead to various diseases (51). A recent study also revealed that excessive NETs can lead to neurological deficits, brain edema, and microcirculatory dysfunction after experimental TBI (13). Circulating NETs biomarkers have been reported to be associated with elevated ICP and worse neurological function in TBI patients (13). Similar results were also detected in our research, in which peripheral plasma NETs, both ds-DNA, and the H3Cit-DNA complex were significantly elevated in TBI patients. Moreover, we also found that neutrophils-derived NETs were significantly increased in brain tissue from TBI patients. Importantly, circulating NETs significantly correlated significantly with elevated ICP and worse neurological function in TBI patients. However, the contribution of NETs to TBI-induced neuronal pyroptotic death and neurological deficits remains to be elucidated. A previous study demonstrated that NET formation was upregulated in brain parenchyma in mice subjected to ischemic stroke and NETs as the main source circulating ds-DNA, could contribute to neuroinflammation and blood-brain barrier disruption *via* the cGAS/STING pathway (33, 37). Notably, STING has been reported to be primarily expressed in neurons and microglia in the brain (52). Consistently, our research found that STING-positive neurons were significantly increased in mice subjected to TBI. In addition, further investigation in our research revealed that degradation of NETs or inhibition of NET formation significantly inhibited STING upregulation in neurons and attenuated neuronal pyroptosis after TBI, whereas overexpression of peptidylarginine deiminase 4 [a key enzyme of NET formation (53)] in the cortex following stereotactic adenoviruses injection could aggravate STING-dependent neuronal pyroptosis. Collectively, these results confirmed that NETs play a detrimental role in promoting neuronal pyroptosis *via* the NETs- STING pathway after TBI.

Severe ER stress is associated with multiple pathophysiological processes of TBI including neuroinflammation, neuronal cell death, blood-brain barrier damage, axon injury, neurodegeneration, etc (45). IRE1α, as one of the vital ER stress sensors, was found to play a critical role in neuronal death, especially in NLRP1 inflammasome-mediated neuronal pyroptosis (30). Consistently, in current experimental research, we found that IRE1α activation was significantly enhanced following TBI, and inhibition of IRE1α effectively rescued NLRP1 inflammasome-mediated neuronal pyroptosis. A recent study reported that NETs could promote intestinal epithelial cell death by regulating ER stress activation-associated protein kinase RNA-like ER kinase (PERK) signaling pathways (31). However, whether and how the NET formation is related to IRE1α activation remains unclear. In the present study, we found that degradation of NETs or inhibition of NET formation significantly inhibited IRE1α activation-associated neuronal pyroptosis after TBI, whereas, overexpression of NETs effectively aggravated IRE1α activation and neuronal death. Importantly, we also explained why NET formation is associated with the activation of IRE1α in the present research. STING, a transmembrane protein located on the endoplasmic reticulum (ER), was demonstrated to act as a bridge between the NETs and IRE1α in our current research.

Several limitations of this study need to be discussed here. First, we did not evaluate the effects of NETs on neural pyroptosis in different age groups or TBI with systemic comorbidities and female animals. Further studies are needed to certify the effect of NETs on neurons in experimental TBI in different age groups and females. Then, in the sample of TBI patients, there was no assessment of the influence of the patient’s operation time and operation area on the experimental results. In addition, in this study, cGAMP, as a product of cGAS, was used as the activator of STING to confirm the role of STING in neurons., However, the role of the cGAS-STING pathway in neuronal pyroptosis caused by NETs is not much confirmed. Further studies are needed to investigate the specific role of cGAS in NETs-mediated STING pathway activation in neurons.

## Conclusions

Our study revealed that NETs were involved in TBI-induced neuronal pyroptosis. It was verified again that the increased NET formation was associated with elevated ICP and worse neurological function in TBI patients. Degradation of NETs or inhibition of NET formation with PAD inhibitor ameliorated TBI-induced IRE1α activation and NLRP1 inflammasome-mediated neuronal pyroptosis, whereas overexpression of NET formation aggravated neuronal pyroptotic death and neurological dysfunction. Moreover, the STING antagonist effectively rescued IRE1α activation and NLRP1 inflammasome-mediated neuronal pyroptosis after TBI. Importantly, the IRE1α antagonist also inhibited NET-induced neuronal pyroptosis and neurological deficits. Collectively, these findings indicated that suppression of the NETs-STING-IRE1α pathway may be protective in TBI-induced NLRP1 inflammasome-mediated neuronal pyroptosis and neurological deficits.

## Data availability statement

The original contributions presented in the study are included in the article/**Supplementary Material**. Further inquiries can be directed to the corresponding authors.

## Ethics statement

The animal study was reviewed and approved by National Institutes of Health Guide for the Care and Use of Laboratory Animals and approved by Tianjin Medical University Animal Care and Use committee. Written informed consent was obtained from the individual(s) for the publication of any potentially identifiable images or data included in this article.

## Author contributions

XC, SZ, JZ designed the experiment. YC MS, LL, XM, and HJ performed most of the experiments. XL, ZC, YuZ, SL, GY assisted in data analysis. QD, FC, XC wrote the manuscript. YaZ supplements the data. SZ, JZ provided the overall guidance. All authors contributed to the article and approved the submitted version.
