# Supplementary material for: Inhibition of neutrophil extracellular trap formation attenuates NLRP1-dependent neuronal pyroptosis via STING/IRE1α pathway after traumatic brain injury in mice
Source: Front Immunol. 2023 Apr 14;14:1125759. doi: 10.3389/fimmu.2023.1125759 (PMC10152368; doi:10.3389/fimmu.2023.1125759)
Supplement: Supplementary file 1 [file DataSheet_1.docx]

Supplementary Material

**Inhibition of neutrophil extracellular trap formation attenuates NLRP1-dependent neuronal pyroptosis** **via STING/IRE1α pathway after traumatic brain injury in mice.**

**Yiyao Cao, Mingming Shi^†^, Liang Liu^†^,** **Yan Zuo****^†^, Haoran Jia,** **Xiaobin Min, Xilei Liu, Zhijuan Chen, Yuan Zhou, Shenghui Li, Guili Yang, Xiao Liu,** **Quanjun Deng,** **Fanglian Chen, Xin Chen^*^,** **Shu Zhang****^*^ and Jianning Zhang^*^**

*** Correspondence:** Xin chen: [xinchentianjin@163.com](mailto:xinchentianjin@163.com); Shu zhang: [zhangshu2017@tmu.edu.cn](mailto:zhangshu2017@tmu.edu.cn); Jianning Zhang: [jianningzhang@hotmail.com](mailto:jianningzhang@hotmail.com)


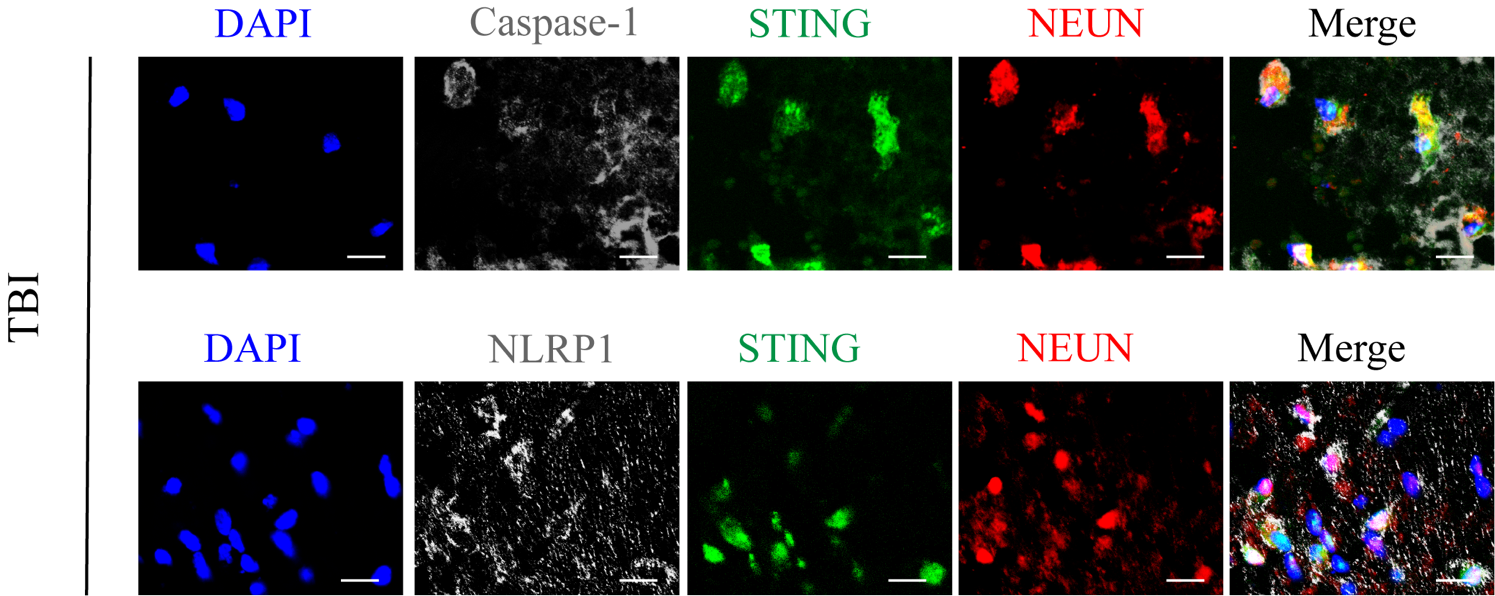


**Supplementary Figure 1.**  Representative fluorescent three-stain image of NEUN-positive(red) neurons with P-STING-positive(green) and NLRP1-positive(gray) cells. Scale bar = 100 μm


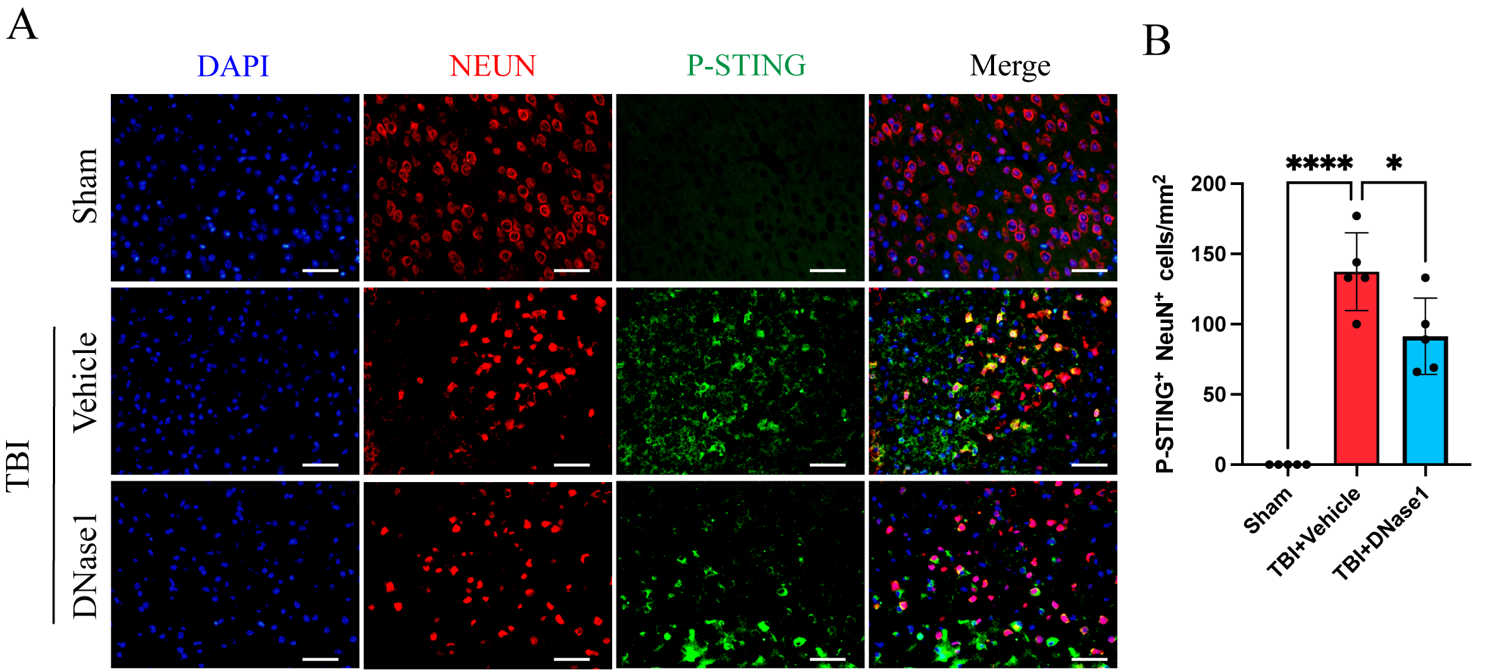


**Supplementary Figure 2.** A Representative fluorescent double-stain image of NEUN-positive(red) neurons with P-STING-positive(green) cells. Scale bar = 50 μm


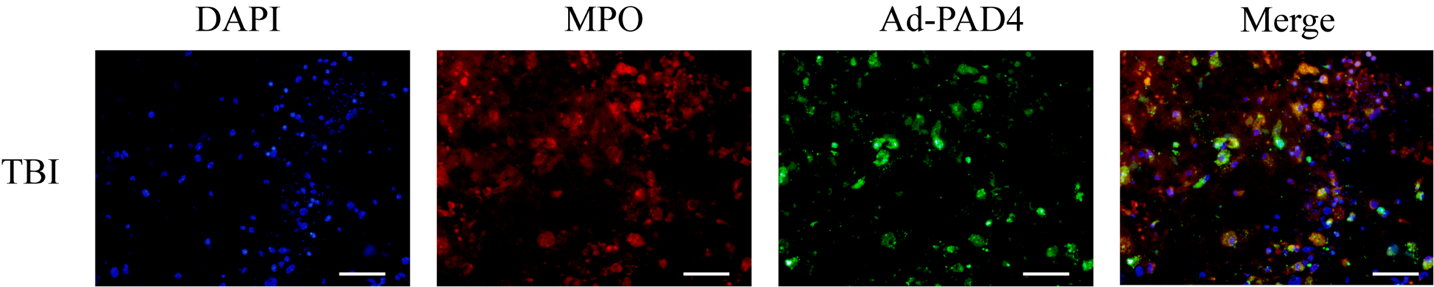


**Supplementary Figure 3.** Representative fluorescent double-stain image of MPO-positive(red) neutrophil with Ad-PAD4-positive(green) cells. Scale bar = 50 μm .

| **Case** | **Age** | **Gender** | **Cause of**  **injury** | **Other**  **injuries** | **Time post-**  **injury (h)** | **Region of**  **surgery** | **GCS** |
| --- | --- | --- | --- | --- | --- | --- | --- |
| Traumatic brain injury | 48 | Male | Traffic accident | None | 12 | Right frontal lobe | 6 |
| Traumatic brain injury | 65 | Male | Traffic accident | None | 7 | Left frontal lobe | 7 |
| Traumatic brain injury | 56 | Female | Traffic accident | None | 11 | Right frontal lobe | 5 |
| Traumatic brain injury | 50 | Female | Traffic accident | None | 8 | Right parietal lobe | 5 |
| Malignant glioma | 43 | Male | - | None | - | Left parietal lobe | - |
| Malignant glioma | 61 | Female | - | None | - | Left parietal lobe | - |
| Malignant glioma | 38 | Male | - | None | - | Right frontal lobe | - |

Table1. Demographics and clinical characteristics of human brain tissues from acute traumatic brain injury and malignant glioma.
